# ALK mutation dynamics and clonal evolution in a neuroblastoma model exhibiting two ALK mutations

**DOI:** 10.18632/oncotarget.27119

**Published:** 2019-08-13

**Authors:** Simon Durand, Cécile Pierre-Eugène, Olivier Mirabeau, Caroline Louis-Brennetot, Valérie Combaret, Léo Colmet-Daage, Orphée Blanchard, Angela Bellini, Estelle Daudigeos-Dubus, Virginie Raynal, Gudrun Schleiermacher, Sylvain Baulande, Olivier Delattre, Isabelle Janoueix-Lerosey

**Affiliations:** ^1^Institut Curie, PSL Research University, Inserm U830, Equipe Labellisée Ligue contre le Cancer, Paris F-75005, France; ^2^SIREDO: Care, Innovation, and Research for Children, Adolescents, and Young Adults with Cancer, Institut Curie, Paris F-75005, France; ^3^Centre Léon Bérard, Laboratoire de Recherche Translationnelle, Lyon F-69008, France; ^4^Equipe SiRIC RTOP (Recherche Translationnelle en Oncologie Pédiatrique), Institut Curie, Paris F-75005, France; ^5^Gustave Roussy, Vectorology and Anticancer Therapies, UMR 8203, CNRS, University Paris-Sud, Université Paris-Saclay, Villejuif F-94805, France; ^6^Institut Curie Genomics of Excellence (ICGex) Platform, Institut Curie Research Center, Paris F-75005, France

**Keywords:** neuroblastoma, ALK mutations, heterogeneity, clonal evolution, ALK inhibitors

## Abstract

The *ALK* gene is a major oncogene of neuroblastoma cases exhibiting ALK activating mutations. Here, we characterized two neuroblastoma cell lines established from a stage 4 patient at diagnosis either from the primary tumor (PT) or from the bone marrow (BM). Both cell lines exhibited similar genomic profiles. All cells in the BM-derived cell line exhibited an ALK F1174L mutation, whereas this mutation was present in only 5% of the cells in the earliest passages of the PT-derived cell line. The BM-derived cell line presented with a higher proliferation rate *in vitro* and injections in Nude mice resulted in tumor formation only for the BM-derived cell line. Next, we observed that the F1174L mutation frequency in the PT-derived cell line increased with successive passages. Further Whole Exome Sequencing revealed a second ALK mutation, L1196M, in this cell line. Digital droplet PCR documented that the allele fractions of both mutations changed upon passages, and that the F1174L mutation reached 50% in late passages, indicating clonal evolution. *In vitro* treatment of the PT-derived cell line exhibiting the F1174L and L1196M mutations with the alectinib inhibitor resulted in an enrichment of the L1196M mutation. Using xenografts, we documented a better efficacy of alectinib compared to crizotinib on tumor growth and an enrichment of the L1196M mutation at the end of both treatments. Finally, single-cell RNA-seq analysis was consistent with both mutations resulting in ALK activation. Altogether, this study provides novel insights into ALK mutation dynamics in a neuroblastoma model harbouring two ALK mutations.

## INTRODUCTION

Neuroblastoma, a cancer of the sympathetic nervous system, is the most frequent extra-cranial solid tumor of childhood [1]. It is characterized by a wide range of clinical heterogeneity with extremely different outcomes between patients with low-risk and high-risk diseases. Long-term survival of high-risk patients still remains inferior to 50% despite complex and aggressive therapies. Ten years ago, the identification of activating ALK mutations in a subset of sporadic and familial neuroblastoma cases [2–5] revealed that ALK might represent a *bona fide* therapeutic target for precision medicine, analogous to other mutated tyrosine kinase receptors involved in various adult cancers. The *ALK* gene encodes one such receptor mostly expressed during the development of the central and peripheral nervous systems [6]. Together with the LTK and ROS receptors, it constitutes a subfamily of the insulin receptor family. Several lines of evidence now suggest that ALK is involved in sympathetic ganglia neurogenesis during normal development [7].

Further large-scale screening studies confirmed that *ALK* mutations occur in approximately 8% of neuroblastoma patients at diagnosis, with three mutation hotspots at residues 1174, 1245 and 1275 [8–11]. High-level *ALK* amplifications have also been reported in 2% of primary tumors [8]. Whereas the initial cohorts consisted of tumors analyzed at diagnosis, subsequent analysis revealed a higher frequency of *ALK* mutations in relapsed disease compared to diagnosis [10, 12, 13]. Several studies documented that the ALK genetic landscape may be even more complicated. Indeed, it has been shown that *ALK* mutations may occur at subclonal levels at both diagnosis and relapse [12, 14]. In a subset of neuroblastoma cases, several *ALK* mutations have been described within the same tumor [2, 3, 15]. Recently, different ALK mutations, R1275L and F1174L, have been identified in a pair of cell lines established at diagnosis and relapse, respectively [16]. These observations may have important consequences in terms of clonal evolution and disease treatment.

The oncogenic properties of the F1174L and R1275Q mutated ALK receptors have been studied in various *in vitro* systems and mouse models [17–20]. These models demonstrated that combined mutated ALK and MYCN signaling (linked to MYCN overexpression) were sufficient to induce neuroblastoma from sympathoadrenal progenitors.

The *ALK* gene is also involved in other types of childhood and adult cancers in which chromosomal rearrangements lead to fusion proteins with constitutive ALK kinase activity (for a review, see [21]). Both ALK-mutated and ALK-rearranged tumors may be targeted by ALK inhibitors, which are competitive inhibitors of the ATP-binding within the kinase domain. The first clinical trial with crizotinib [22] in children suggested that inhibition of mutated ALK in neuroblastoma may be more difficult to achieve compared to inhibition of ALK fusions such as the NPM-ALK fusion in anaplastic large-cell lymphomas [23]. In neuroblastoma, different ALK mutations have been shown to exhibit different activation levels and response to crizotinib [8]. In particular, a partial resistance to crizotinib was reported for the ALK F1174L mutated receptor. Alectinib has been shown to exert antitumor activity not only on EML4-ALK and NPM-ALK fusions but also on EML4-ALK bearing a L1196M mutation, described as a gatekeeper mutation conferring resistance to ALK inhibitors [24].

In this work, we characterized two neuroblastoma cell lines derived from the same patient at diagnosis exhibiting either the ALK F1174L mutation at a clonal level or both the ALK F1174L and L1196M mutations at different frequencies. We took advantage of this cellular model to investigate ALK mutation dynamics, sensitivity to crizotinib and alectinib, and transcriptomic profiles at the single-cell level.

## RESULTS

### Characterization of isogenic neuroblastoma cell lines with different ALK mutation status

**Figure 1:**
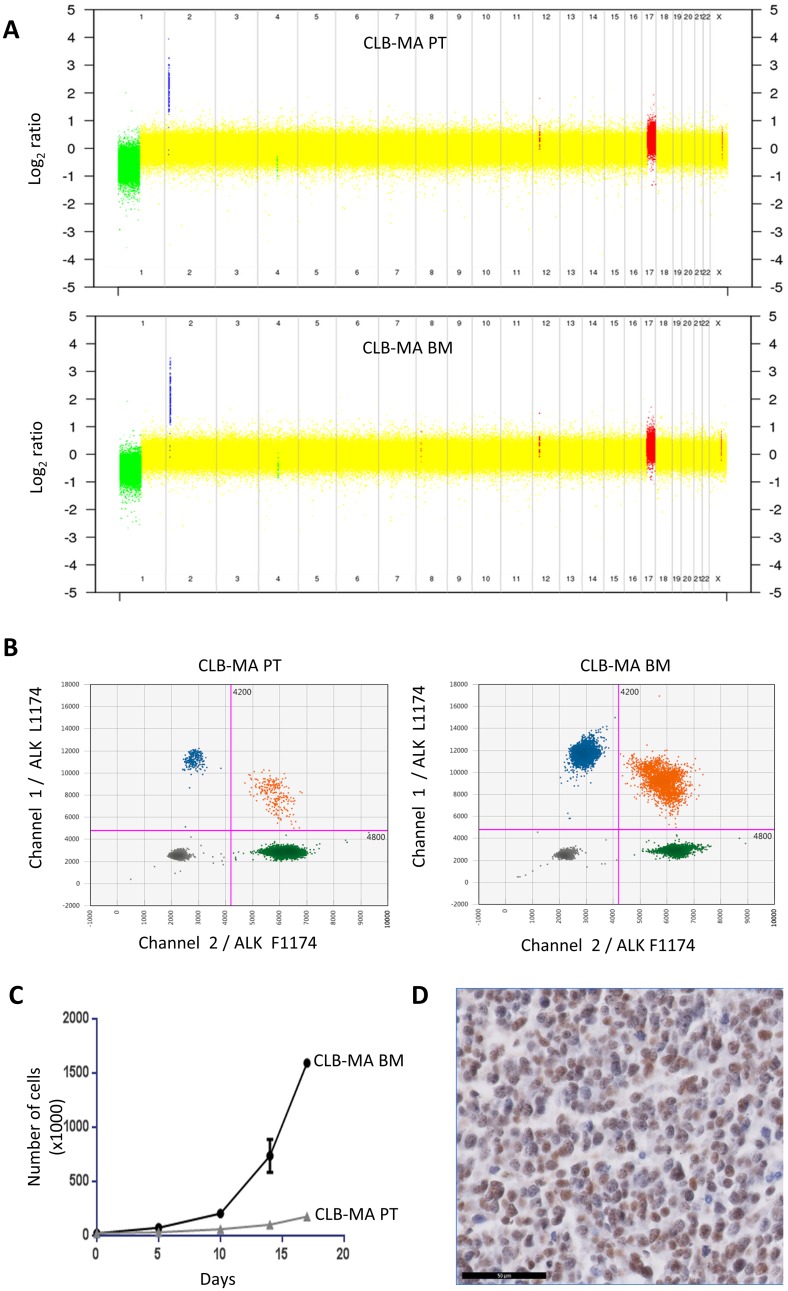
Genomic alterations and growth of the CLB-MA PT and CLB-MA BM neuroblastoma cell lines. (**A**) The copy number profile obtained on CytoScan^®^ HD arrays shows *MYCN* amplification (blue), 1p loss (green) and 17q gain (red) in both cell lines. (**B**) Droplet digital PCR (ddPCR) indicates a fraction of 2.5% of the ALK F1174L mutation in the CLB-MA-PT cell line whereas this mutation is present at a fraction of 49.6% in the CLB-MA-BM cell line. Green and blue dots represent droplets containing only ALK F1174 or ALK L1174 sequences, respectively. Orange dots correspond to mixed droplets containing both the ALK WT F1174 and the ALK mutated L1174 sequences. Grey dots correspond to empty droplets. (**C**) *In vitro* proliferation of CLB-MA BM and CLB-MA PT cells at passage 6. 20,000 cells were plated in 12-well plates and cells were counted at days 5, 10, 14 and 17. (**D**) PHOX2B immunohistochemistry performed on an orthotopic xenograft of CLB-MA BM cell line. Scale bar: 50 μm.

In this work, we focused on two neuroblastoma cell lines established from a female stage 4 neuroblastoma patient at diagnosis, either from the primary abdominal tumor (CLB-MA PT) or from the invaded bone marrow (CLB-MA BM). The CLB-MA BM cell line is identical to the cell line called CLB-MA used in our previous publications [4, 14, 25]. For clarity and comparison with the CLB-MA PT cell line, we refer to it as CLB-MA BM in this study. Both cell lines have been established before any treatment. Figure 1A shows that they share a similar copy number profile, with 1p loss, 17q gain and *MYCN* amplification. Both samples exhibit two copies of the *ALK* gene (Supplementary Figure 1). The CLB-MA BM presents with a clonal ALK F1174L (c.3522C>A) mutation, that had been previously detected by Sanger sequencing [4] and was measured here by droplet digital PCR (ddPCR) with an allele fraction of 50% (Figure 1B). This indicates that all cells exhibit one copy of the mutation. Interestingly, ddPCR for the CLB-MA PT sample revealed the same ALK F1174L mutation but being subclonal with an allele fraction of 2.4% at passage 3 (Figure 1B). Deep sequencing using MiSeq confirmed this fraction of mutated allele (data not shown), which likely corresponds to 5% of the cells bearing a heterozygous mutation.

We then measured the proliferation capacity of both cell lines *in vitro*. As shown in Figure 1C, the CLB-MA BM cell line exhibited a higher proliferation rate compared to the CLB-MA PT cell line (used at passage 6), consistent with a growth advantage associated with the *ALK* mutation. Then, orthotopic xenografts were performed for both cell lines in the adrenal gland of Nude mice. Tumor growth was observed for the CLB-MA BM cell line in 3 out of 3 mice whereas no tumor growth could be detected for the CLB-MA PT cell line (passage 7) in 2 injected mice. PHOX2B immunohistochemistry confirmed that the obtained tumors were neuroblastomas from the injected CLB-MA BM cell line (Figure 1D).

### Dynamics of ALK mutated clones in the CLB-MA PT cell line

**Figure 2:**
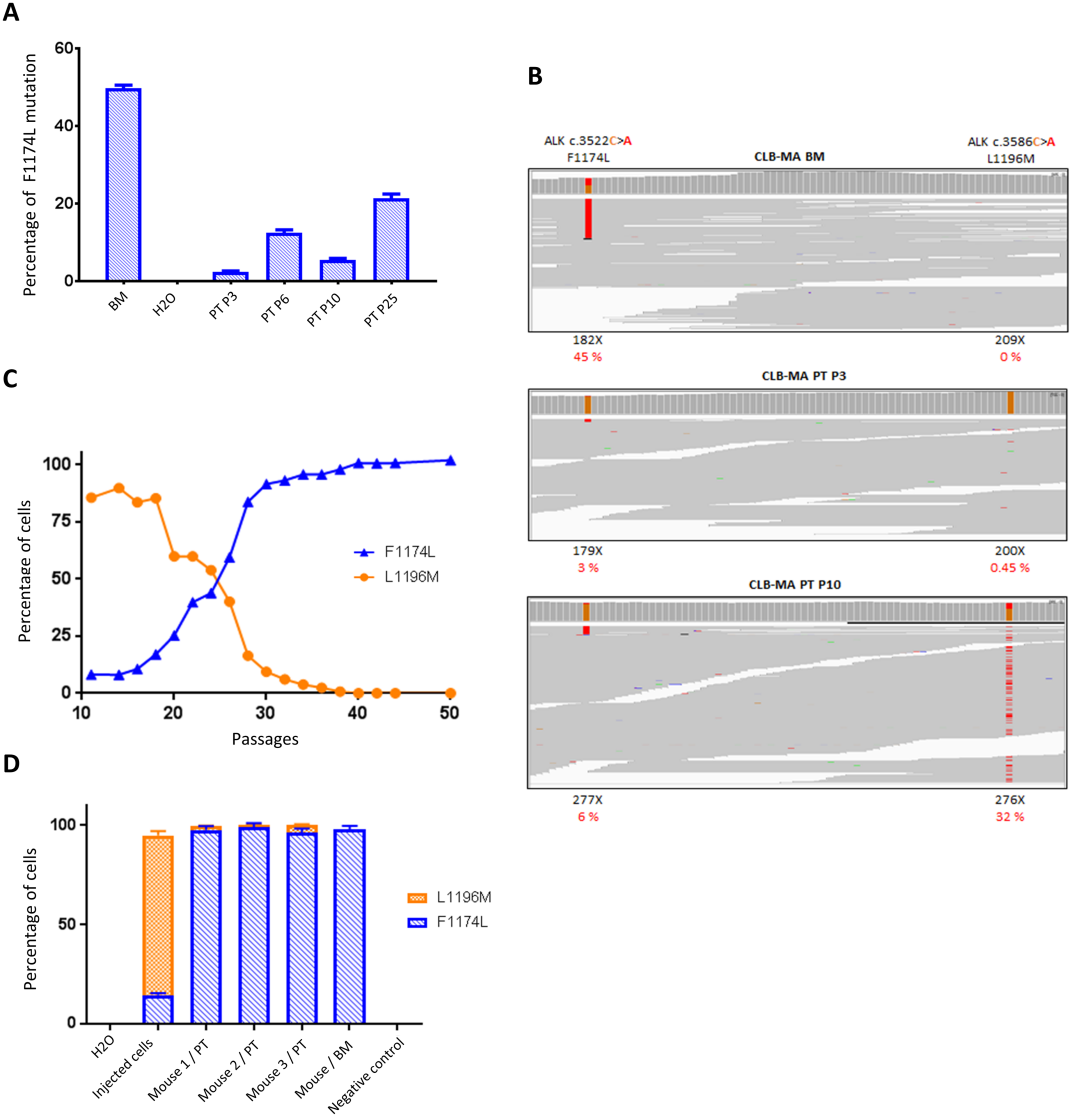
Identification of a second ALK mutation (L1196M) in CLB-MA PT and dynamics of subclone growth *in vitro* and *in vivo*. (**A**) Increase of ALK F1174L mutated allele fraction determined by ddPCR in CLB-MA PT cell line through successive passages *in vitro*. The CLB-MA BM is shown on the left. Each sample was processed in duplicate. (**B**) IGV profiles of the F1174 and L1196 positions obtained by Whole Exome Sequencing of the CLB-MA BM, CLB-MA PT at passages 3 and 10. X: total number of reads for the indicated position. In red, the percentage of alternative reads supporting the variation among the total number of reads. Data in (A) and (B) were obtained with the first batch of CLB-MA PT cells. (**C**) The percentages of cells bearing the F1174L (blue) and L1196M (orange) mutations in the CLB-MA PT cell line from passage 11 to passage 50 of the second batch have been determined by ddPCR. (**D**) Enrichment of the F1174L mutation in tumors obtained from orthotopic xenografts of the CLB-MA PT cell line injected at passage 14 (second batch). The tumor of mouse 1 was harvested 3 months after injection whereas tumors of mice 2 and 3 were harvested 2.5 months after injection. The respective percentages of cells with each of the two mutations are shown. DNA extracted from mouse muscle was used as a negative control. Each sample was processed in duplicate.

Since we observed a subclonal ALK F1174L mutation in the PT-derived cell line analyzed shortly after cell line establishment, we sought to determine whether this mutation could be enriched across time in culture. We therefore cultured the CLB-MA PT cell line and measured the mutated allele fraction by ddPCR from passage 3 to passage 25. We observed that the F1174L allele fraction increased with successive passages, reaching more than 20% at passage 25 (Figure 2A), which would correspond to 40% of the cells bearing the ALK F1174L mutation provided that this mutation is heterozygous. To further characterize these clones and explore their dynamics of growth, we performed WES of the CLB-MA PT cell line, at different passages. Consistently with the ddPCR results, the WES approach allowed to detect a F1174L mutated allele fraction of 3.35, 9.73 and 6.14% at passages 3, 6 and 10, respectively (Figure 2B and Table 1). WES of the CLB-MA BM cell line revealed an ALK F1174L allele fraction of 45%. Surprisingly, our WES detected a second ALK L1196M (c.3586C>A) mutation in the CLB-MA PT cell line, which was observed at 0.45% at passage 3 and increased dramatically at passages 6 and 10 to reach an allele fraction of 23.5% and 32.2%, respectively (Table 1). No read for the ALK L1196M mutation could be detected in the CLB-MA BM cell line.

**Table 1:** Frequencies of ALK mutations detected by ddPCR and WES in the various CLB-MA cell lines

Cell line	Passage number	% F1174L (ddPCR)	% F1174L (WES)	% L1196M (ddPCR)	% L1196M (WES)
CLB-MA BM	-	49.6	45.05	0	0
CLB-MA PT P3	3	2.38	3.35	0.54	0.45
CLB-MA PT P6	6	12.4	9.73	24.1	23.51
CLB-MA PT P10	10	5.49	6.14	32.6	32.25

In order to confirm our results and further increase the number of passages, we performed a second set of experiments starting from a vial of cells frozen at passage 8 in the first experiment shown in Figure 2A. Due to the dynamics of each mutation, the F1174L and L1196M allele fractions were systematically monitored by ddPCR at each step of the subsequent experiments performed with this second batch. Figure 2C shows that the L1196M was enriched in early passages (85% of cells bearing this mutation at P11). Then, cells with the ALK F1174L mutation likely grew at a higher rate. At passage 24, our data are consistent with a situation in which one-half of the cells exhibits the ALK F1174L and the other half exhibits the L1196M mutation. The L1196M mutation was no more detectable in late passages (>40), in which the F1174L mutated allele fraction reached 50%, indicating that the cell population contains almost exclusively ALK F1174L mutated cells. Both the F1174L and L1196M mutations are located within the kinase domain of the receptor and have been shown to be activating [8]. They are observed in the Phe core and active site of the receptor, respectively, and the L1196M mutation has been described as a gatekeeper mutation in NPM-ALK [26].

Analysis of WES data revealed a total of 33 variations observed in at least one of the 4 analyzed samples (Supplementary Table 1). Only the two ALK mutations were associated with a specific ID in the COSMIC cancer database. We observed variants predicted to be deleterious with the 4 prediction tools used (described in the materials and methods section) in the *IGF1R* and *ARHGAP6* genes. However, the observed variation in IGFR1 is outside the kinase domain and does not affect critical phosphorylation sites. The ARHGAP6 variation is different from the ones previously reported in neuroblastoma [11].

We next further characterized the growth properties and oncogenic potential of the different mutated clones of the CLB-MA PT cell line. First, to explore the link between the proliferation rate and ALK mutations, we measured the proliferation capacity of the CLB-MA PT at passages P12, P19 and P27. As shown in Supplementary Figure 2, cell numbers increased more rapidly according to the passage of the cell line (P27>P19>P12) consistently with their ALK F1174L allele frequency measured by ddPCR, respectively of 54%, 36% and 4.5% at the time of plating. In addition, since cells of the first batch of this cell line were not able to grow in Nude mice, we performed orthotopic injection of cells (passage 14 of the second batch) in 3 NSG mice, characterized by a more immunocompromised phenotype compared to Nude mice. Tumors developed in the 3 injected mice and we next determined the allele fractions of both F1174L and ALK L1196M mutations in the obtained tumors by ddPCR. As shown in Figure 2D, whereas the F1174L mutation was present in around 14% of the injected cells, the percentage of cells with this mutation was close to 100% in the three obtained tumors, strongly suggesting a selection of the cells displaying a growth advantage. This *in vivo* observation is in agreement with the clonal selection of the ALK F1174L mutated subclone observed *in vitro*, and its enrichment in late passages during cell culture.

### Impact of ALK inhibitors on the growth of F1174L and L1196M mutated clones

**Figure 3:**
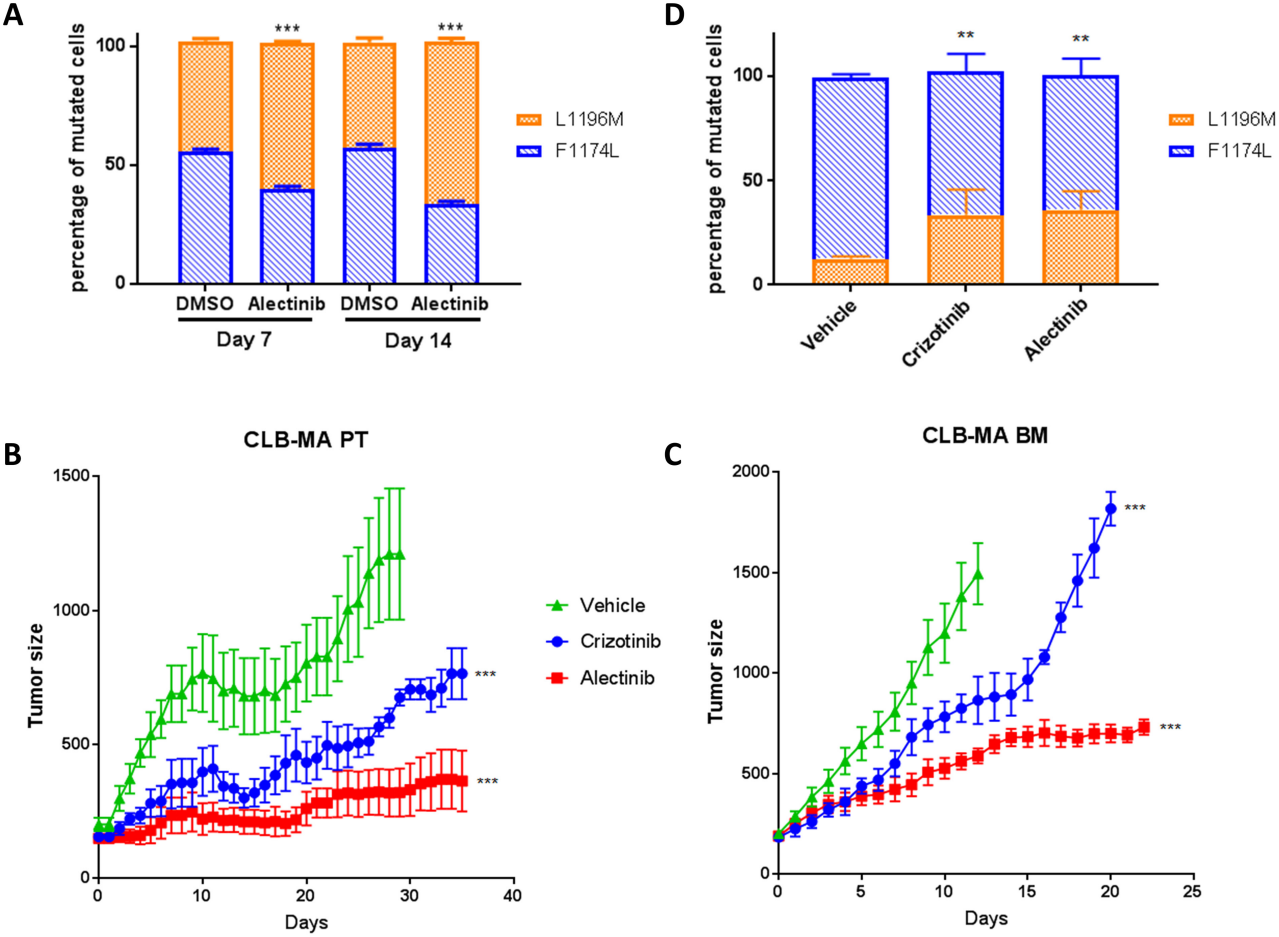
Sensitivity of ALK F1174L and L1196M mutations to ALK inhibitors *in vitro* and *in vivo*. (**A**) Enrichment of the ALK L1196M mutation upon alectinib treatment *in vitro*. The respective percentages of F1174L (blue) and L1196M (orange) mutated cells after *in vitro* treatment of the CLB-MA PT cell line at passage 25 with alectinib (0.5 μM) for 7 or 14 days was determined by ddPCR. Five biological replicates were analyzed in each condition, and each sample was processed in duplicate. Statistical difference was evaluated using a nonparametric Mann-Withney test. (**B**) Effect of the ALK inhibitors alectinib and crizotinib on the growth of subcutaneous xenografts of the CLB-MA PT cell line injected at passage 14 (second batch). Mice were treated 6 days per week with vehicle (*n* = 5), crizotinib at 100 mg/kg/day (*n* = 5) or alectinib at 60 mg/kg/day (*n* = 5) when tumor volumes were around 200 mm^3^. Mice were sacrificed after 35 days of treatment. Mean tumor volume (+/– s.d.) is shown. (**C**) Effect of the same inhibitors on the growth of subcutaneous xenografts of the CLB-MA BM cell line; vehicle (*n* = 5), crizotinib (*n* = 3) or alectinib (*n* = 8). Mice were sacrificed when tumor volume reached 2000 mm^3^. Mean tumor volume (+/– s.d.) is shown until time of sacrifice of the first mouse of each group (day 13 after treatment’s start for the control group; day 21 for crizotinib). In (B) and (C) the statistical difference between each inhibitor and the vehicle was assessed for the whole curve using a nonparametric Wilcoxon test. (**D**) Respective percentages of ALK F1174L (blue) and L1196M (orange) mutated cells in tumors obtained from CLB-MA PT cells after 35 days of treatment with crizotinib or alectinib or vehicle. Five biological replicates were analyzed in each condition, and each sample was processed in duplicate. Statistical difference was evaluated using a nonparametric Mann-Withney test. ^**^ *p*-value < 0.01; ^***^ *p*-value < 0.001.

Importantly, differences in sensitivity to various ALK small-molecule inhibitors have been reported for different ALK mutants [8, 27]. The ALK inhibitor alectinib (CH5424802) has been shown to be able to block the resistant gatekeeper mutation L1196M [24]. To explore the behavior of the F1174L and L1196M-mutated clones in the CLB-MA PT cell line during long-term treatment, we sought to treat these cells *in vitro* with low doses of alectinib. In the control samples without treatment, after 7 and 14 days of culture, the percentages of F1174L and L1196M mutated cells measured by ddPCR were 55% and 45%, respectively (Figure 3A). Treatment with 0.5 μM of alectinib resulted in an enrichment of the L1196M-mutated cells in both conditions compared to DMSO treatment. As shown in Supplementary Figure 2, an enrichment of the ALK L1196M mutation upon 6 or 8 days of alectinib treatment was also observed when using the CLB-MA PT cell line containing a higher proportion of F1174L-mutated cells. In addition, crizotinib treatment resulted as well in an enrichment of L1196M-mutated cells. Short treatments of 24 hours with both inhibitors did not modify the proportion of each mutation compared with the control condition (Supplementary Figure 3).

We then evaluated the efficacy of the ALK inhibitors crizotinib and alectinib on mouse xenografts of the CLB-MA PT and CLB-MA BM cell lines. In both cases, our data show that alectinib is more efficient to impair tumor growth compared to crizotinib (Figure 3B and 3C). For the CLB-MA PT xenografts, analysis of the allele fraction of each ALK mutation after 35 days of crizotinib or alectinib treatment revealed an enrichment of the ALK L1196M mutated cells in the remaining tumors compared to the vehicle-treated tumors (Figure 3D). These observations therefore indicate that the ALK L1196M mutation is more resistant to both ALK inhibitors than the F1174L mutation, both *in vitro* and *in vivo*.

### Transcriptomic analysis of the isogenic cell lines at the single-cell level

**Figure 4:**
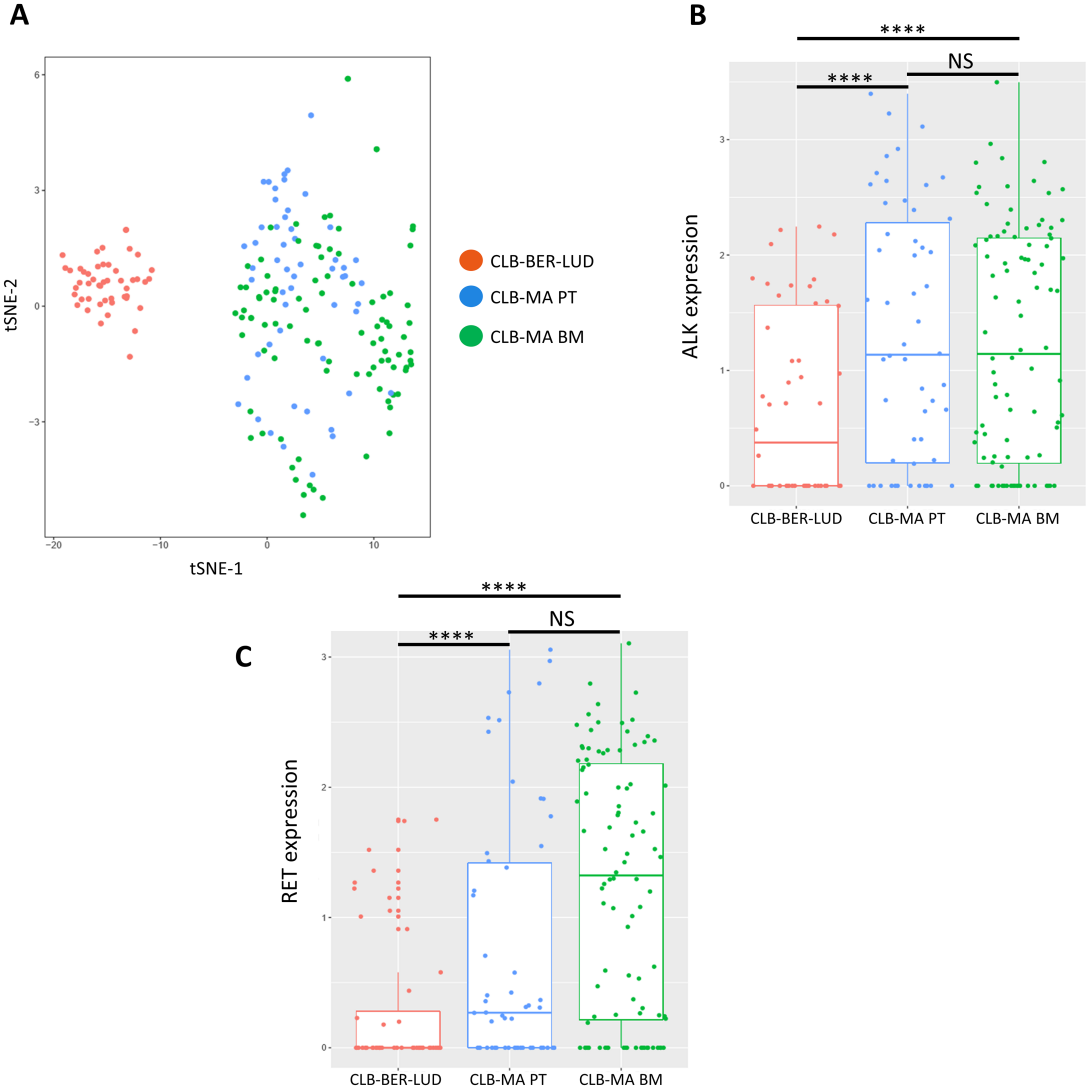
Single-cell RNA-seq analysis on CLB-MA PT and CLB-MA BM cell lines. (**A**) t-SNE analysis performed on RNA-seq data generated for 58 CLB-MA PT cells, 96 CLB-MA BM cells, and 48 CLB-BER-LUD (ALK WT) cells. (**B**) and (**C**) ALK and RET expression level are shown for the 3 cell lines, respectively.

To further characterize the CLB-MA PT and CLB-MA BM cell lines, we performed single-cell RNA seq analysis of 58 and 96 cells of each cell line, respectively. The CLB-MA PT cell line contained around 8% of F1174L-mutated cells and 90% of L1196M mutated-cells at the time of this experiment. We included in the analysis 48 cells of the CLB-BER-LUD cell line, another neuroblastoma cell line derived from a stage 4 patient with *MYCN* amplification but no *ALK* mutation. Whereas CLB-MA BM and CLB-MA PT cells were grouped together in a t-SNE (t-Distributed Stochastic Neighbor Embedding) analysis, CLB-BER-LUD cells appeared as a distinct group (Figure 4A). The ALK expression level measured at the single-cell level in the three analyzed cell lines (Figure 4B) is consistent with the previous description of an increased ALK expression in neuroblastoma samples with activated ALK compared to samples with WT ALK [18, 28, 29]. However, we could not determine the ALK mutation status in each individual cell since the number of reads on exon 23 was too low in most cases. The *RET* gene previously described as a target of activated ALK at the transcriptional level [18, 25, 30] also showed an increased expression in CLB-MA PT and CLB-MA BM cells compared to CLB-BER-LUD cells (Figure 4C). Although a trend suggesting a higher expression of RET in CLB-MA BM cells compared to CLB-MA PT cells was observed, the difference in the median values was not statistically significant. Next, we performed a differential expression analysis to compare the CLB-MA BM and CLB-MA PT cell lines transcriptomic profiles (Supplementary Table 2). Among the 314 differentially expressed genes, 288 and 26 genes were up-regulated or down-regulated in CLB-MA BM cells compared to CLB-MA PT cells, respectively. Gene Ontology analysis of the two gene sets revealed no significant enrichment in categories related to molecular function, biological process or cellular component. Altogether these data are consistent with both the ALK F1174L and L1196M mutations being activating and suggest that only very subtle differences may be observed in the transcriptomic profiles of individual cells bearing one or the other mutation. Further combined RNA-seq and DNA-seq analysis will be required to determine the ALK mutation status and the corresponding transcriptomic profile in a sole cell.

## DISCUSSION

Whereas a couple of neuroblastoma cell lines have been generated from the same patient at diagnosis and relapse (such as SK-N-BE(1) and SK-N-BE(2), SMS-KCN and SMS-KCNR or NBLW or NBLW-R) [16, 31], our study took advantage of a unique model of two neuroblastoma cell lines derived from the same patient at diagnosis from the primary tumor or from a metastatic site, before any treatment. We used this pair of cell lines to explore the dynamics of ALK mutations and sensitivity to various ALK inhibitors. Indeed, these samples represented isogenic cell lines showing the same genetic alterations, including 1p loss, 17q gain and *MYCN* amplification, which are emblematic of neuroblastoma, and differed only by their ALK status. Interestingly, the CLB-MA BM cell line derived from the patient’s bone marrow exhibited an F1174L mutation likely present in each tumor cell. Indeed, we measured an allele fraction for this mutation of 50% and 45% by ddPCR and WES, respectively, and SNP analysis documented two copies of the ALK locus. In contrast, the combined analysis of the CLB-MA PT cell line using ddPCR and WES revealed that this cell line harbored two different ALK mutations, F1174L and L1196M, whose proportions varied during cell culture. At the earliest passages (first cell batch), the F1174L and L1196M mutations were both observed at a low subclonal level. The L1196M showed a rapid enrichment during early passages for the second cell batch, which is likely due to a selection of cells bearing this mutation upon thawing. Then at passage 24, a similar allele fraction of both mutations was observed; at late passages, the L1196M mutation was no more detectable and the F1174L allele fraction reached 50%. Since we could never detect any reads bearing both mutations in the WES data and since the cumulated frequency of both mutations never exceeded 100%, the likely hypothesis is that the two mutations are contained in two distinct cellular clones. In this work, we also performed WES for the primary tumor itself, exhibiting a tumor cell content of 50%. A unique read was observed bearing the F1174L mutation among more than 300 reads covering the position of interest (data not shown). Therefore, we cannot exclude that the ALK F1174L mutation was present in the primary tumor at a very low frequency and that this mutation has been positively selected during the establishment of the CLB-MA PT cell line.

The comparison of the growth properties of both cell lines revealed that the CLB-MA BM presented with a higher proliferation rate and was more prone to develop tumors in Nude mice compared with the CLB-MA PT cell line at early passages (P6 of the first batch). This is consistent with the strong oncogenic activity of the ALK F1174L mutation, previously demonstrated *in vitro* and *in vivo* [17–20]. Furthermore, we observed a preferential growth of ALK F1174L-mutated cells from the CLB-MA PT cell line *in vivo* in NSG mice, although ALK L1196M-mutated cells were present in a much higher proportion in the injected cells. These *in vivo* observations highly suggest that the L1196M mutation present with a more modest constitutive activation compared to the F1174L mutation, as previously described *in vitro* [8]. Importantly, in a subset of neuroblastoma patients, it has been documented that ALK activating mutations may be subclonal at diagnosis and at relapse [13, 14]. This suggests that, at least in some patient tumors, ALK-mutated cells do not proliferate as a single clone and that potential cooperation between ALK-mutated and ALK-wild-type cells may be important for tumor growth and fitness. The cooperation of different tumor cell subpopulations have already been described in different adult cancers and it has been documented that even rare tumor subclones may be of crucial importance in the whole tumor growth [32, 33].

Interestingly, it has been documented that various ALK mutations exhibit different sensitivity to various ALK inhibitors. A reduced sensitivity has been reported for the ALK F1174L mutated receptor towards the first-generation ALK inhibitor crizotinib [8, 34]. Furthermore, both mutations have been described as acquired crizotinib resistance mutation in tumors driven by ALK fusion proteins, including non-small-cell lung carcinoma (NSCLC) and inflammatory myofibroblastic tumors [35–37]. The ALK F1174L mutant receptor has been shown to exhibit an increased ATP-binding affinity, which consequently reduces the efficacy of the ATP competitive small-molecule inhibitor [34]. This may potentially be reversed by increasing the doses of the inhibitor. The ALK L1196M is a gatekeeper mutation also exhibiting crizotinib resistance. This mutation in the full-length ALK receptor corresponds to the L256M mutation in NPM-ALK originally described as a mutation resistant to several compounds acting as kinase inhibitors and relates to the Thr 314 and Thr 790 positions in the ABL and EGFR receptors, respectively [26]. Mutations of these gatekeeper residues have been shown to promote the assembly of an enzymatically active kinase conformation through the stabilization of a series of hydrophobic interactions [38]. Next-generation ALK inhibitors have been designed to bypass such resistance mechanisms. Ceritinib is able to overcome resistance to the L1196M but not to the ALK F1174L mutation [39]. Alectinib has been described as an inhibitor blocking the resistance gatekeeper mutation in the EML4-ALK fusion, *i.e.* efficient on an EML4-ALK L1196M protein [24]. We therefore explored the dynamics of ALK mutations in the CLB-MA PT cell line upon treatment with either crizotinib or alectinib. Using sub-lethal doses of the inhibitor to avoid massive cell death, we first documented an enrichment of the ALK L1196M *in vitro*. We then demonstrated a better therapeutic efficiency of the alectinib compared to crizotinib. Nevertheless, enrichment of the L1196M mutant was also observed after *in vivo* treatment. These observations suggest that the L1196M mutant receptor is more resistant to these inhibitors compared to the ALK F1174L mutated form and that the proportions of ALK mutated clones may be affected by small-molecule inhibitors.

Recently, multiple acquired resistance mutations in the ALK receptor have been reported after sequential use of several ALK inhibitors to treat a patient with an ALK-rearranged advanced NSCLC [40]. Multiple point mutations developed after treatment with crizotinib (L1196M), belizatinib (F1174L and G1269A) and ceritinib (F1174L, D1203K and G1269A). Of note, the ALK L1196M mutation that first appeared as a resistance mutation to crizotinib treatment was not detected anymore in the subsequent biopsy specimens analyzed along treatment. Interestingly, the third-generation ALK inhibitor lorlatinib has been shown to be active against all single ALK resistance mutations occurring in the EML4-ALK fusion [41]. In neuroblastoma, lorlatinib has demonstrated promising results as a single agent in various pre-clinical models, being able to overcome crizotinib resistance and showing dramatic inhibition of tumor growth of neuroblastoma xenografts or MYCN and ALK^F1174L^ driven tumors in a transgenic mouse model [42, 43]. However, sequential treatment of ALK-positive lung cancer with several ALK inhibitors including lorlatinib can select for compound ALK mutations with high-level resistance [44]. These data suggest that more efficacious ALK-targeted therapy may rely on the use of a third-generation inhibitor as an up-front treatment to prevent emergence of resistance mutations in ALK-rearranged lung carcinoma. Lessons learned from the use of ALK inhibitors in adult cancers may certainly be a benefit in the setup of ALK therapy for neuroblastoma patients.

In conclusion, our work illustrates the complexity of the ALK mutation status in a subset of neuroblastoma cases and demonstrates the need for inhibitors able to counteract all activating mutations. It also highlights the importance of studying pre-clinical models that reproduce intra-tumor heterogeneity observed in patient tumors.

## MATERIALS AND METHODS

### Cell culture, DNA extraction, and cell proliferation assays

The CLB-MA PT, CLB-MA BM and CLB-BER-LUD neuroblastoma cell lines were cultured in RPMI supplemented with 10% FBS (Eurobio) and 100 μg/ml penicillin-streptomycin (Gibco) at 37°C with 5% CO_2_ in a humidified atmosphere. Cells were routinely checked by PCR for the absence of mycoplasma. When reaching 90% of confluency, cells were detached using TrypLE™ Express Enzyme (ThermoFischer) and diluted at 1:3. STR profiling confirmed that the CLB-MA PT and CLB-MA BM cell lines have been derived from the same patient.

For DNA extraction, cells were washed twice with PBS and 2.10^6^ cells were lysed and DNA was extracted using the QIAamp DNA mini kit (Qiagen). For cell proliferation assay, cells were plated in 12-well plates and the number of viable cells was determined in triplicates using the Vi-CELL XR counting machine (Beckman Coulter).

### Array-CGH

Affymetrix genome-wide human CytoScan^®^ HD arrays were used to obtain whole genomic profiles according to Affymetrix recommendations*.*

### Mouse xenografts

Orthotopic injection of 10^6^ cells in the left adrenal gland was done by a left subcostal incision under ketamine/xylazine anesthesia in Nude or NSG mice (Charles River). Mice were monitored and euthanized at the onset of clinical signs or if a large tumor was detected by palpation. At sacrifice, tumors were processed for IHC or flash-frozen in liquid nitrogen. Half of the frozen tumor was crushed and DNA was extracted using the QIAamp kit (Qiagen).

Subcutaneous xenografts of 10.10^6^ cells suspended in 50% DMEM-50% matrigel were performed in the right flank of NSG (CLB-MA PT) or SCID (CLB-MA BM) mice. Tumor size was measured 6 days per week. When tumors reached 7 mm of diameter, mice were randomly attributed to one of the 3 groups of treatment (see below). Animal care and use for this study were performed in accordance with the recommendations of the European Community (2010/63/UE) for the care and use of laboratory animals. Experimental procedures were specifically approved by the ethics committee of the Institut Curie CEEA-IC #118 (Authorization APAFIS#11206-2017090816044613-v2 given by National Authority) in compliance with the international guidelines.

### Digital droplet PCR

Each reaction was performed using 50 ng of DNA and the ddPCR™ Supermix for Probes (No dUTP, Bio-Rad Technologies) in a final volume of 20 µL. Each of the two probes was added to obtain a 250 nM final concentration and the primers were used at 450 nM. This 20 µL solution was combined to 70 μL of Droplet Generation Oil (Bio-Rad Technologies) and used for droplet generation on the QX200™ machine (following manufacturer’s general guidelines). Next, the PCR on the generated droplets was performed on the CFX96 Touch™ Real-Time PCR Detection System as follows: 95°C for 10 min, 40 cycles at 95°C for 30 sec and 55°C for 1 min and finally 98°C for 10 min. Droplets were then transferred to a QX100 digital droplet reader (Bio-Rad Technologies) to measure fluorescent intensities of FAM and VIC probes. Primers and probes used are described in Table 2. Results were analyzed using QuantaSoft Software (Bio-Rad Technologies). Each experiment included a non-template, a negative and a positive control. Each sample was processed in duplicate.

**Table 2:** Primers and probes used for the detection of ALK F1174, L1174, L1196 and M1196 alleles by ddPCR

Name	Type	Sequence	Target
ALK_3522_for	primer	CCAATGCAGCGAACAA	ALK exon 23
ALK_3522_rev	primer	TTTGGTTACATCCCTCTCT	ALK exon 23
ALKwt_3522C	probe	Yakima yellow -TGGTGGTTGAATTTGCTGC-MGB	F1174
ALKmut_3522A	probe	6FAM -TGGTGGTTTAATTTGCTGC-MGB	L1174
ALK_3586_for	primer	TTTGGTTACATCCCTCTCTGC	ALK exon 23
ALK_3586_rev	primer	GACTGGTTCTCACTCACCG	ALK exon 23
ALKwt_3586C	probe	Yakima yellow -TTCATCCTGCTGGAGCTCATGG-MGB	L1196
ALKmut_3586A	probe	6FAM-TTCATCCTGATGGAGCTCATGG-MGB	M1196

### Immunohistochemistry

Mice tumors were dissected at sacrifice and fixed with acidified formal alcohol (AFA) for 24 h and paraffin-embedded. Labeling was performed on 4 µm sections with the Lab Vision IHC stainer Autostainer 480 (Thermo Scientific). Briefly, sections were deparaffinized, antigen retrieval was performed using citrate buffer at pH 6.1, and sections were stained with a mouse monoclonal anti-PHOX2B antibody (Santa Cruz, sc-376997) at 1/200.

### Whole Exome Sequencing (WES)

WES was performed using the Nextera Exome kit (Illumina) for CLB-MA BM and the corresponding germline DNA or using the SureSelect Clinical Research Exome V1 (Agilent Technologies) for the CLB-MA PT cell line at passages 3, 6 and 10 and for the primary tumor of the patient. Exome enrichment kits were used according to the manufacturer’s protocols. WES was performed using Illumina Hi-Seq2000 (CLB-MA BM and germline) or Hi-Seq2500 (CLB-MA PT cell line samples) leading to paired-ends 100 x 100 bp. Sequencing was done to obtain an expected coverage above 100x. Following alignment with Bowtie2 [45] allowing up to 4% of mismatches, bam files were cleaned according to the Genome Analysis Toolkit recommendations [46]. WES variant calling was performed using GenomeAnalysisTK-3.5 HaplotypeCaller. Annovar-v2013-07-29 with cosmic-v64, dbsnp-v137, and RefSeq were used for annotations, and functional prediction was performed using Polyphen2, LRT, MutationTaster and Sift. SNVs with a quality <30, a depth of coverage <20 in tumor, or <2 reads supporting the variant were filtered out. Only SNVs within exons of coding genes or splice sites were kept. Then, variants reported in more than 1% of the population in the 1000 genomes (1000gAprl_2012), or Exome Sequencing Project (ESP6500) or Exome Aggregation Consortium (ExAC03) were discarded to filter out polymorphisms. Finally, synonymous variants were filtered out except those with a COSMIC ID. Variant calling comparison between germline and somatic DNAs allowed us to focus only on tumor-specific SNVs. Only nongermline SNVs supported by>2 reads and a position coverage >20x were taken into account as tumor-specific alterations.

### ALK inhibitor treatments

Crizotinib was purchased from MedChemExpress. Alectinib obtained from Roche/Chugai was used to treat mice with subcutaneous xenografts of CLB-MA BM cell line. Alectinib purchased from Selleckchem was used for all other experiments. For *in vitro* treatments, the medium and the ALK inhibitors were replaced every two days. Alectinib and crizotinib were dissolved in 100% dimethyl sulfoxide (DMSO) to reach a stock concentration of 1 mM and 55 mM, respectively, and stored at –20°C before use.

For *in vivo* treatments, alectinib was diluted at 6 mg/mL in a vehicle solution of 10% DMSO, 10% cremophor, 15% polyethylene glycol 400 (PEG 400), 15% hydroxypropyl-β-cyclodextrin (HPCD) and 0.02N Hydrochloric acid (HCl). Crizotinib was diluted in 50% DMSO. Mice were weighed and treated by oral gavage with 60 mg/kg/day for the alectinib-treated group and the equivalent volume for the vehicle group, and with 100 mg/kg/day for the crizotinib-treated groups.

### Single-cell transcriptomic analysis

Single cells loading, capture and mRNA pre-amplification were performed following the Fluidigm user manual “Using C1 to Generate Single-Cell cDNA Libraries for mRNA Sequencing”. Briefly, adherent cells were detached using TrypLE™ Express Enzyme (ThermoFischer), 600 cells were loaded into a medium-cell integrated fluidic circuit (Fluidigm) and captured in the Fluidigm C1 System. The capture efficiency was assessed by imaging with a microscope. Lysis, reverse transcription and preamplification were done using with the SMARTer Ultra Low RNA Kit (Clontech) in the Fluidigm C1 System. The amplified cDNA was harvested the following day. cDNA quantity and quality were assessed using picogreen Qubit^®^ fluorometer (Thermo Fischer) and Bioanalyzer instrument (Agilent). Libraries were obtained using 0.6 ng of preamplified cDNA using Nextera XT DNA Library prep kit. Sequencing was done on the HiSeq 2500 System (Illumina).

Reads obtained from sequencing of cells were aligned on the human genome (v. hg19) using TopHat (version 2.0.6) [47]. Reads mapping more than once (parameter -x1) or having edit distances of more than 3 (-N3) were discarded. Counting of reads on annotated genes from the GRCh37 gene build was done using htseq-count (v. HTSeq-0.5.3p9) [48] with the following parameters: reads with a quality score less than 10 (-a 10) were discarded and reads partially overlapping with the annotated gene transcript were included in the counts unless they overlapped with another read. The STRANDED=no option was used in all experiments. Sample-to-sample normalization was performed by rescaling using DESeq size factors [49].

## SUPPLEMENTARY MATERIALS






