# Supplementary material for: ALK mutation dynamics and clonal evolution in a neuroblastoma model exhibiting two ALK mutations
Source: Oncotarget. 2019 Aug 13;10(48):4937–50. doi: 10.18632/oncotarget.27119 (PMC6697636; doi:10.18632/oncotarget.27119)
Supplement: Supplementary file 1 [file oncotarget-10-4937-s001.pdf]

ALK mutation dynamics and clonal evolution in a neuroblastoma model exhibiting two ALK mutations

SUPPLEMENTARY MATERIALS

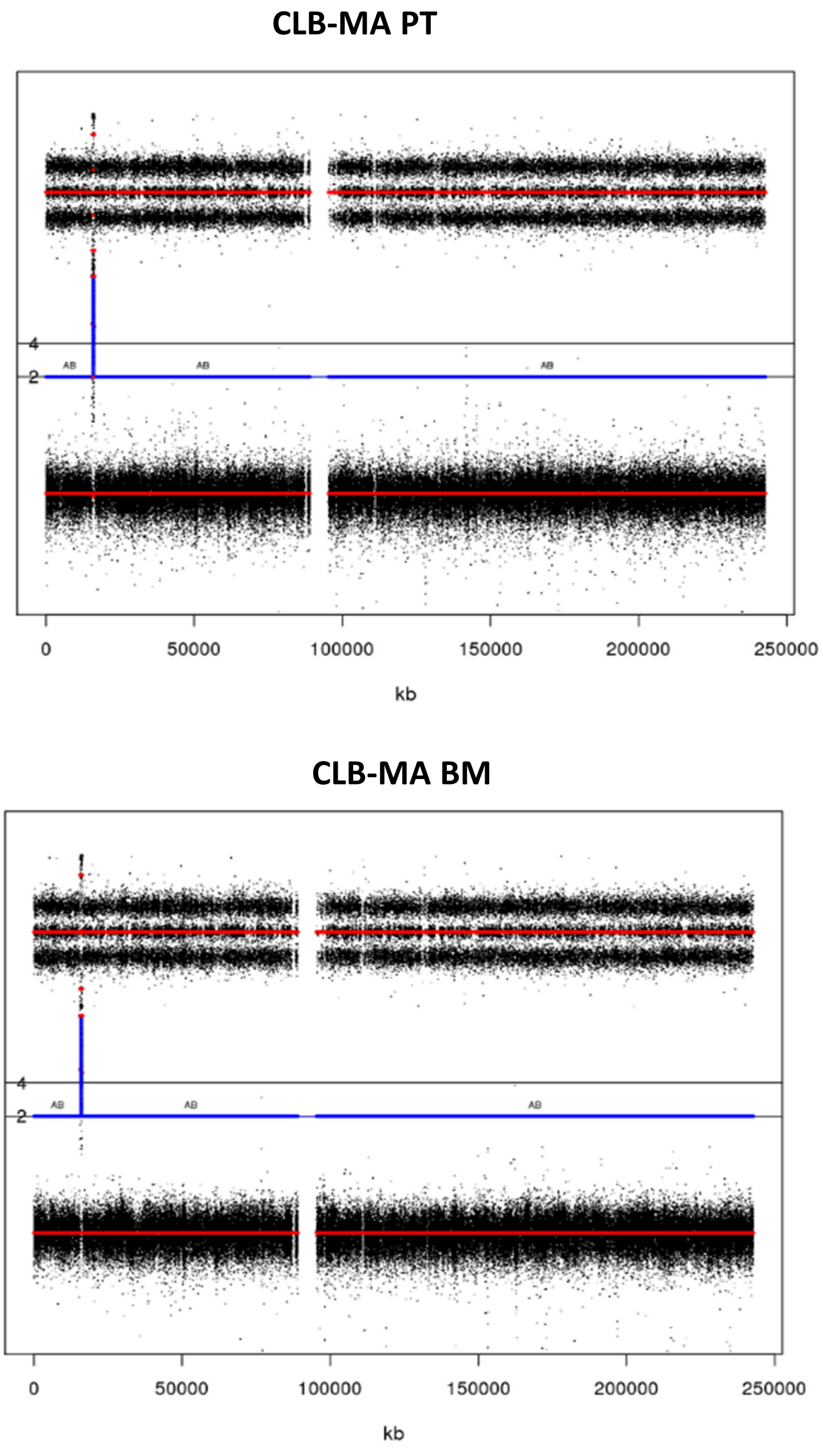

Supplementary Figure 1: B allele frequency (BAF) and copy number obtained from the CytoScan® HD arrays are shown along chromosome 2 for the CLB-MA BM and CLB-MA PT cell lines.

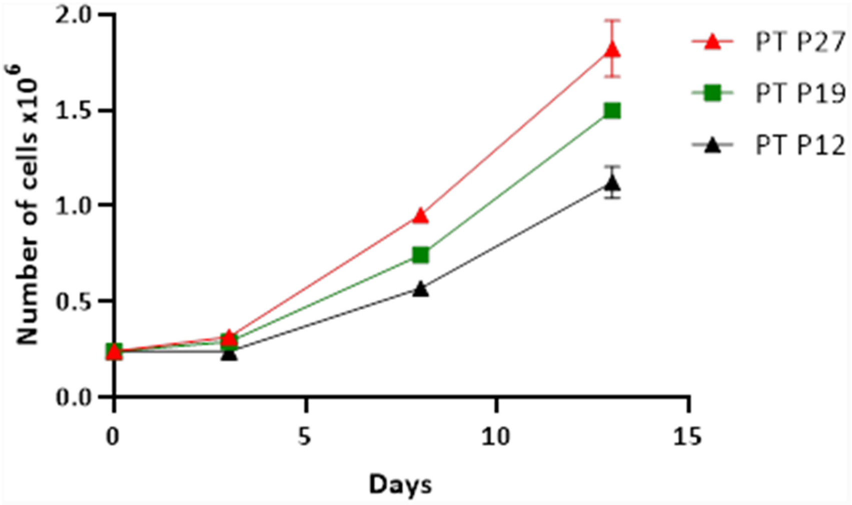

Supplementary Figure 2: ***In vitro*** **proliferation of the CLB-MA PT cell line at passages 12, 19 and 27.** 240,000 cells were plated in 12-well plates and cells were counted at days 3, 8 and 13.

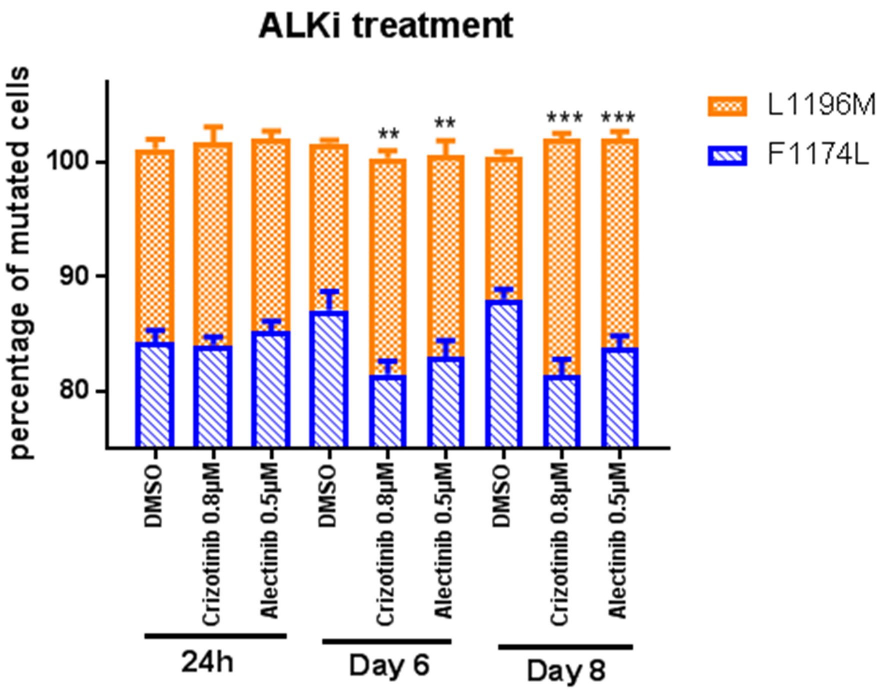

Supplementary Figure 3: The percentages of L1196M and F1174L mutated cells after ***in vitro*** **treatment of the CLB-MA PT cell line with alectinib (0.5 µM) or crizotinib (0.8 µM) for 24 hours, 6 or 8 days were determined by ddPCR.** Three biological replicates were analyzed in each condition, and each sample was processed in duplicate.

Supplementary Table 1: Other SNVs detected by WES in the various CLB-MA cell lines. See Supplementary_Table_1

Supplementary Table 2: Results of the differential analysis of RNA-seq data comparing 58 CLB-MA PT cells with 96 CLB-MA BM cells. Data normalization and differential analysis were performed using the DESeq software. See Supplementary_Table_2
